# Effects of Plant and Animal Natural Products on Mitophagy

**DOI:** 10.1155/2020/6969402

**Published:** 2020-03-10

**Authors:** Farzaneh Shakeri, Vanessa Bianconi, Matteo Pirro, Amirhossein Sahebkar

**Affiliations:** ^1^Natural Products and Medicinal Plants Research Center, North Khorasan University of Medical Sciences, Bojnurd, Iran; ^2^Department of Physiology, School of Medicine, North Khorasan University of Medical Sciences, Bojnurd, Iran; ^3^Unit of Internal Medicine, Angiology and Arteriosclerosis Diseases, Department of Medicine, University of Perugia, Perugia, Italy; ^4^Halal Research Center of IRI, FDA, Tehran, Iran; ^5^Biotechnology Research Center, Pharmaceutical Technology Institute, Mashhad University of Medical Sciences, Mashhad, Iran; ^6^Neurogenic Inflammation Research Center, Mashhad University of Medical Sciences, Mashhad, Iran

## Abstract

Mitophagy is a protected cellular process that is essential for autophagic removal of damaged mitochondria and for preservation of a healthy mitochondrial population. In the last years, a particular interest has been devoted in studying the effects of natural compounds on mitophagy. Different natural compounds may modulate mitochondrial oxidative phosphorylation, the production of mitochondrial reactive oxygen species, the expression of mitophagy- and autophagy-related genes, and the activities of transcription factors which regulate the expression of mitochondrial proteins, thereby controlling mitochondrial damage and mitophagy. Remarkably, since mitochondrial function has a crucial role in the pathogenesis of various diseases (e.g., cancer, atherosclerosis, Duchenne muscular dystrophy, diabetes complications, Alzheimer's disease, and hepatic steatosis), these effects might have important therapeutic implications. In this review, preclinical studies investigating the role of different natural compounds in the modulation of mitophagy will be discussed.

## 1. Introduction

**Figure 1:**
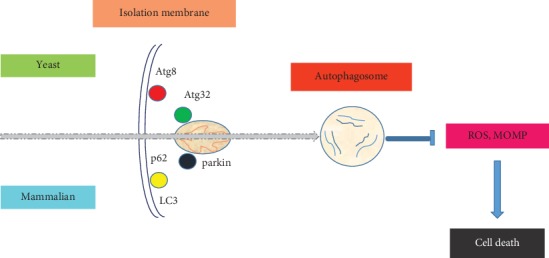
Schematic representation of mitophagy. ROS: reactive oxygen species; MOMP: mitochondrial outer membrane permeabilization.

Mitochondria, double membrane-enclosed organelles of eukaryotic cells, have a crucial role in the regulation of cellular energy homeostasis and cell death [1]. Mitophagy, which was first described over one hundred years ago [2], is the mechanism by which impaired or superfluous mitochondria are engulfed in autophagosomes to be then degraded into lysosomes [3, 4]. Mitophagy is critical for the maintenance of proper cellular functions. Removal of damaged mitochondria through mitophagy requires two steps: induction of general autophagy and priming of damaged mitochondria for selective autophagic recognition [5, 6]. Several protein receptors, including autophagy-related protein (Atg)32 in yeast, Nix/BCL2 interacting protein 3 like (Bnip3l), BCL2 interacting protein 3 (Bnip3), and FUN14 domain containing 1 (Fundc1) in mammalian systems, directly act in mitophagy. Atg32 interacts with Atg8 on the surface of mitochondria, promoting core Atg protein assembly for mitophagy. Nix/Bnip3l, Bnip3, and Fundc1 also have a classic motif to directly bind to microtubule-associated protein 1A/1B light chain 3 (LC3) (Atg8 homolog in mammals) for activation of mitophagy (Figure 1) [3].

In the last decades, a particular interest has been devoted in studying the effects of different natural compounds of vegetable and animal origin, often found in dietary products at biologically active concentrations, on the modulation of pathophysiological processes underlying different diseases [7]. More recently, some of these natural compounds have attracted attention for their ability to modulate mitochondrial homeostasis. In this review, we will discuss recent findings from experimental studies concerning the effects of different natural compounds, including resveratrol, curcumin, *E. uniflora*, *G. formosana*, berberine, *P. americana*, *P. suffruticosa*, quercetin, quercetogetin, Shanxi aged vinegar (SAV), sulforaphene, tomatidine, and toxicarioside H, on mitophagy (Table 1).

## 2. Methods

We searched the literature available in ISI Web of Knowledge, Medline, PubMed, Scopus, and Google Scholar databases for English articles published until January 2019. For this purpose, we used appropriate keywords such as “mitophagy,” “natural compound,” “plants,” “phytochemical,” and “nutraceutical.” Twenty-four studies were considered eligible for inclusion in this review (Table 1). Abstracts, unpublished articles, and non-English language articles were excluded.

## 3. Gastrointestinal Disease

The intestinal epithelium is one of the most rapidly self-renewing tissues and needs a great amount of energy. In intestinal epithelial cells, a hyperstimulation of mitochondria-mediated production of cellular energy may occur, leading to the overproduction of ROS. An excessive ROS production, in turn, may promote mitochondrial damage, disruption of the oxidation respiratory chain, and activation of cell death pathways. Therefore, mitophagy, which can mediate the clearance of damaged mitochondria before they cause activation of cell death, is crucial for the homeostasis of the intestinal epithelium [8].

Resveratrol (3,5,4′-trihydroxy-trans-stilbene) is a natural polyphenol belonging to the phytoalexin family [9]. This substance has different biological properties including antitumor [10], antioxidant [11], antiviral [12], and phytoestrogenic [13] effects.

Cao et al. investigated the effects of dietary administration of resveratrol (100 mg/kg) on mitochondrial dysfunction and mitophagy in intestinal mucosa epithelial cells of diquat-challenged piglets [8]. They showed that resveratrol reduced diquat-induced mitochondrial ultrastructure abnormalities (e.g., swelling and vacuolation) and ameliorated mitochondrial function of intestinal mucosa epithelial cells by inducing mitophagy. Accordingly, resveratrol increased phosphatase and tensin homolog (PTEN), induced putative kinase 1 (PINK1) and Parkin expression levels, decreased the generation of reactive oxygen species (ROS), and increased the mitochondrial membrane potential, the content of mitochondrial DNA (mtDNA), and the activity of mitochondrial complexes I–IV.

In a study by Martins et al., treatment with resveratrol (1, 10, and 50 *μ*M, for 24 h) increased activated hepatic stellate cell (i.e., GRX cell) death signals by altering mitochondrial dynamics and function. In fact, all resveratrol concentrations stimulated autophagosome formation by increasing the expression levels of autophagy-related proteins [14].

Wu et al. investigated the effects of resveratrol on mitochondrial ROS production and NLR family pyrin domain containing 3 (NLRP3) inflammasome activation in high glucose containing peritoneal dialysis (PD) solution-treated human peritoneal mesothelial cells (HPMCs). In their study, treatment with resveratrol (0, 25, and 50 *μ*mol/l, for 48 h) induced mitophagy through the adenosine monophosphate-activated protein kinase (AMPK) activation and protected HPMCs from oxidative stress and NLRP3-mediated inflammatory injury [15].

*Eugenia uniflora* (*E. uniflora*), also called pitanga or Brazilian cherry [16] or Cerisier Carré [17], is a plant of the Myrtaceae family, native of the east coast of tropical South America. Different pharmacological properties of this plant, such as gastroprotective [18], anti-inflammatory, antioxidant [19], antinociceptive [20], and antibacterial [21] effects, have been reported. The effect of ethanolic *E. uniflora* extract (5, 50, and 100 *μ*g/ml, for 72 h) on autophagy was evaluated in GRX cells (a well-established line) by Denardin et al. [22]. In their study, *E. uniflora* extract increased the expression activated hepatic stellate cell levels of autophagy mediators such as Atg7. In addition, results of flow cytometry and ultrastructural analyses of treated cells indicated that the number of mature autophagosomes and autolysosomes significantly increased after treatment with *E. uniflora* extract [22].

Quercetin is a natural flavonoid found abundantly in apples, honey, raspberries, onions, red grapes, cherries, citrus fruits, and green leafy vegetables [23]. There is growing evidence suggesting that quercetin has therapeutic potential for the treatment of different diseases, including cardiovascular diseases [24], cancer [25], and neurodegenerative diseases [26]. The effects of quercetin on chronic ethanol-induced hepatic mitochondrial damage in mice were investigated by Yu et al. [27]. In their study, oral quercetin administration (100 mg/kg, for 15 weeks) reduced hepatocyte damage and mitochondrial morphological abnormalities (e.g., fractured endoplasmic reticulum, lipid droplets near the mitochondria, swelling, and restructuring of mitochondrial inner membranes) and dysfunction. In addition, quercetin inhibited ethanol-induced mitophagy suppression by increasing the expression levels of mitophagy mediators, such as LC3-II, p62, the mitochondrial outer membrane protein required for Parkin-dependent mitophagy voltage-dependent anion-selective channel 1 (VDAC1), Parkin, and FoxO3a, and by decreasing the expression levels of ubiquitin-specific protease 30 (Usp30), an inhibitor of Parkin-mediated mitophagy. In addition, quercetin induced mitophagy through the upregulation of the AMPK and extracellular signal-regulated kinase 2 (ERK2) signaling pathways [27].

In a study by Liu et al., the effects of oral quercetin administration (100 mg/kg, for 10 weeks) on high-fat diet- (HFD-) induced hepatic steatosis in mice were evaluated. Treatment with quercetin reduced HFD-induced body weight gain and disorders of lipoprotein metabolism by reducing the expression of lipogenic genes, such as fatty acid synthase (FAS), and by increasing the expression of carnitine palmitoyltransferase I (CPT1), a key enzyme in fatty acid *β*-oxidation. These quercetin-mediated beneficial effects against hepatic steatosis were in part dependent on a reduced mitochondrial damage and increased mitophagy activation. In fact, quercetin upregulated the expression of mitophagy mediators (e.g., Parkin, PINK1, Bnip3, Fundc1, LC3-II, p62, Beclin-1, and frataxin) and improved Parkin translocation to mitochondria. In addition, quercetin promoted mitophagy through the stimulation of frataxin-mediated activation of PINK1/Parkin-dependent mitophagy [28].

Shanxi aged vinegar is a traditional Chinese rice vinegar produced by spontaneous solid-state fermentation [29]. Several SAV-mediated pharmacological effects have been described such as antioxidant [30], hypotensive, hypoglycemic, and cholesterol-lowering effects [31]. The protective effects of SAV extract against bromobenzene- (BB-) induced hepatotoxicity were investigated by Yang et al. In their study, SAV extract inhibited hepatocyte ROS production and induced hepatocyte mitophagy by increasing the expression levels of autophagy mediators such as LC3-II, Beclin-1, and p62 through the activation of the protein phosphatase 2A- (PP2A-) Akt signaling pathway [32].

## 4. Musculoskeletal Disease

Duchenne muscular dystrophy (DMD) is a severe type of muscular dystrophy in humans characterized by progressive weakness of skeletal muscles and cardiomyopathy. DMD is caused by genetic mutations of the dystrophin gene. The absence of dystrophin causes intracellular Ca^2+^ dysregulation, resulting in mitochondrial dysfunction and increased production of ROS [33].

The effects of resveratrol on cardiomyocyte mitochondrial damage and autophagy of damaged mitochondria in the *mdx* mouse (a Duchenne muscular dystrophy model) heart were evaluated by Kuno et al. [33]. In their study, oral administration of resveratrol (0, 5, 50, and 500 mg/kg, for 56 weeks) ameliorated the cardiomyopathy by reducing the amount of mtDNA deletion, ROS production by damaged mitochondria, and the number of mitochondria-containing autophagosomes. Such effects were mediated by resveratrol-induced accumulation of the nuclear transcription factor Forkhead box O3 (FoxO3a) and FoxO-induced expression of autophagy-related genes [33].

In a similar study, Sebori et al. investigated the protective effects of resveratrol in skeletal muscle cells of the *mdx* mice [34]. In their study, oral administration of resveratrol (0.04, 0.4, and 4 g/kg, for 56 weeks) reduced myofiber wasting and promoted skeletal muscle cells maturation, leading to a significant reduction of the number of immature myofibers and to an increased number of thicker myofibers in the quadriceps. Accordingly, resveratrol reduced the creatine kinase levels and improved the animals' physical performance in rotarod and four-limb hanging tests. An increased clearance of damaged mitochondria probably contributed to these beneficial effects as resveratrol significantly increased the expression levels of mitophagy- and autophagy-related genes such as *PINK1*, *Parkin*, *sequestosome-1* (*SQSTM1*), *Bnip3*, *Fundc1*, *Atg5*, *Beclin-1* (*Becn1*), *microtubule-associated proteins 1A/1B light chain 3B* (*Map1lc3b*), *transcription factor EB* (*Tfeb*), and *lysosomal-associated membrane protein 1* (*Lamp1*) [34].

## 5. Cancer

Recent studies have shown that the expression of a number of proteins involved in the mitophagy processes, including MFN1, Parkin, BNIP3, and BNIP3L/NIX, is dysregulated in different cancer types [35]. However, the role of mitophagy in tumor development and progression remains largely unclear.

The anticancer effects of resveratrol (0.001-1000 *μ*M, for 48 h) on HeLa cells were investigated by Rodríguez-Enríquez et al. [35]. In their study, biochemical mechanisms underlying resveratrol-induced inhibition of HeLa cell growth and promotion of HeLa cell death were an excessive cellular ROS production corresponding with a significant decrement in the superoxide dismutase (SOD) activity and glutathione (GSH) levels, a decreased oxidative phosphorylation, and a strong mitophagy activation [35].

Curcumin is a naturally occurring phenolic compound obtained from *Curcuma longa*. Several curcumin-mediated pharmacological effects have been described, including anti-inflammatory [36–38], immunomodulatory [39], antitumor and chemosensitizing [40–42], anti-ischemic [43], hepatoprotective [44], relaxant [45], and antiasthmatic effects [46, 47]. Effects of curcumin (10 *μ*M) on the viability of nasopharyngeal carcinoma CNE2 cells exposed to ultrasound were investigated by Wang et al. In their study, ultrastructure of mitochondria was disrupted and mitophagy was induced by curcumin in CNE2 cells [48].

Effects of curcumin (25 *μ*M, for 24 h) on apoptosis of human hepatoma-derived Huh-7 cells were investigated by Moustapha et al. In these cells, curcumin promoted the formation of autophagic vacuoles containing degraded mitochondria and induced autophagy by increasing the expression levels of LC3-II [49].

In a study by Maiti et al., the effects of curcumin or solid lipid curcumin particles (SLCP) (25 *μ*M for 24 h) on autophagic responses were evaluated in cultured U-87MG, GL261, F98, C6-glioma, and N2a cells. Treatment with curcumin or SLCP increased the expression levels of autophagy markers such as Atg5, Atg7, Beclin-1, LC3, and p62 and decreased the expression levels of mitophagy markers such as Fundc1, Bnip3, PINK1, and hypoxia-inducible factor 1-alpha (HIF-1*α*) by inhibiting the phosphoinositide 3-kinase (PI3K)/protein kinase b (Akt)/mammalian target of rapamycin (mTOR) pathway. In addition, cell survival markers were downregulated and cell death markers were upregulated by curcumin. All these effects were amplified in SLCP-treated cells in comparison to curcumin-treated cells [50].

*Gynura formosana* (*G. formosana*), a herbal medicine belonging to the Compositae family, is widely cultivated in the north, south, and east coasts of Taiwan. The pharmacological properties of *G. formosana* include hypoglycemic [51], anti-inflammatory, antioxidant [52], and antibacterial activities [53]. The cytostatic effect of ethyl acetate extract of *G. formosana* (0, 15, 30, 45, 60, 75, 90, and 105 *μ*g/ml, for 72 h) on different tumor cell lines, including HeLa (cervical cancer), HepG2 (liver cancer), and MCF7 (breast cancer) cells, was investigated by Ma et al. [54]. In their study, *G. formosana* significantly decreased cell viability by inducing autophagic flux. Accordingly, the clearance of p62, a common autophagic substrate, and the conversion of LC3-I to LC3-II were enhanced in a time- and dose-dependent manner [54].

*Paeonia suffruticosa* (*P. suffruticosa*), which belongs to the Paeoniaceae family, has been widely used in traditional Chinese medicine for the treatment of various pathological conditions, including macula, epilepsy, and menstrual disorders [55]. Several pharmacological effects can be mediated by *P. suffruticosa*, including antispasmodic, antidiabetic [56], anti-inflammatory [57], antioxidant, anticancer [58], and antimelanogenic effects [59]. The effects of *P. suffruticosa* aqueous extracts on the survival, proliferation, and migration of pancreatic cancer (PC) cells (i.e., PANC1, AsPC1, and BxPC3) were evaluated by Liu et al. [60]. Treatment with *P. suffruticosa* (25–2500 *μ*g/ml, for 24 h) decreased cell survival in a dose-dependent manner through an increased expression of LC3-II, a reduced expression of MFN2 and MFN1, and the promotion of mitophagy through an increased autophagosome and autolysosome formation. Additionally, *P. suffruticosa* inhibited cell cycle progression and cell migration through the downregulation of cyclin and cyclin-dependent kinase (CDK) and the stabilization of F-actin cytoskeleton [60].

Sulforaphene is a natural isothiocyanate extracted from *Raphanus sativus*, a medicinal herb used for over a thousand years in traditional Chinese medicine. Several sulforaphene-mediated pharmacological effects have been described such as anti-inflammatory [61], antioxidant [62], antiasthmatic [63], and anticonvulsant effects [64]. In a study by Wang et al., treatment with sulforaphene (0-20 *μ*M, for 72 h) induced apoptosis and cell cycle arrest in human lymphoma cell lines, such as U937, HUT78, Raji, JeKo-1, and U2932, by triggering simultaneous mitophagic cell death. Sulforaphene-induced mitophagy occurred through the chromosomal maintenance 1- (CRM1-) mediated p62 overexpression and AMPK activation. In fact, different autophagy-related genes such as *SQSTM1*, *valosin-containing protein* (*VCP*), and *apoptosis regulator Bcl-2* (*BCL2*) were expressed after sulforaphene treatment and a time-dependent elevation of different autophagy mediators was seen in the total cell lysate [65].

Toxicarioside H is a cardenolide extracted from the seeds of the tropical medicinal plant *Antiaris toxicaria* [66] and is commonly used in the treatment of congestive heart failure and arrhythmias [67]. Recently, toxicarioside H has been shown to block tumor cell proliferation and induce tumor cell apoptosis through the regulation of different signaling pathways [68]. In a study by Huang et al., treatment with toxicarioside H (0-0.8 *μ*M, for 24 h) was reported to inhibit the proliferation of human lung cancer cell line (i.e., A549 and H460) by promoting mitochondrion-mediated apoptosis. However, in the same study, toxicarioside H was also reported to exert a cytoprotective effect by inducing mitophagy through the upregulation of SIRT3 and the increased interaction of Parkin with VDAC1 [69]. Therefore, toxicarioside H induced lung cancer cell damage and mitochondrion-mediated apoptosis, but it simultaneously allowed lung cancer cells to counteract mitochondrion-mediated apoptosis. Accordingly, the inhibition of toxicarioside H-induced mitophagy by siSIRT3 interference resulted in a more significant toxicarioside H-mediated proapoptotic effect.

## 6. Diabetes

Mitochondria not only play an important role in cellular respiration and ROS production but also have a crucial role in the glucose metabolism of some cells, including myocytes. In patients with diabetes, skeletal muscle dysfunction due to mitochondrial deficits may occur, leading to reduced muscle strength. Particularly, mitochondrial homeostasis and mitochondrial quality control are essential for the maintenance of muscle mass since they indirectly control myocyte insulin sensitivity [70].

Wang et al. evaluated the effects of resveratrol on muscle atrophy in streptozocin-induced diabetic mice. Dietary administration of resveratrol (a diet containing 0.04% of resveratrol, for 8 weeks) improved muscle function in rip strength and treadmill running tests. Such effects were associated with an increased mitochondrial biogenesis and a reduced mitophagy activation in skeletal muscle cells. Accordingly, resveratrol reduced the expression of two muscle-specific E3 ubiquitin ligases, that is, muscle atrophy F-box (MAFbx)/atrogin-1 and muscle RING-finger protein-1 (MuRF-1), and the expression of LC3-II and cleaved caspase-3. In addition, resveratrol increased the expression of nuclear respiratory factor- (NRF-) 1, cytochrome c oxidase (Cox) IV, peroxisome proliferator-activated receptor gamma coactivator 1-alpha (PGC-1*α*), and mitochondrial transcription factor A (mtTFA) and reduced the expression of mitochondrial fission regulatory proteins (e.g., phospho-dynamin-related protein 1 (p-DRP1), mitochondrial fission 1 protein (FIS1), and mitochondrial fission factor (MFF)) and mitochondrial fusion regulatory proteins (e.g., p-DRP1 (Ser637), mitofusin-2 (MFN2), and OPA1 mitochondrial dynamin like GTPase (OPA1)) [70].

## 7. Alzheimer's Disease

Alzheimer's disease is an irreversible, progressive brain disorder, which is characterized by the development of amyloid plaques and neurofibrillary tangles consisting of aggregated *β*-amyloid and tau, respectively. Recent studies indicated that *β*-amyloid could induce mitochondrial abnormalities via disrupting electron transfer chain, increasing ROS production, and impairing mitochondrial function [71].

The effect of resveratrol on mitophagy in amyloid beta-peptide_1-42_- (A*β*_1–42_-) treated PC12 cells, an in vitro model of Alzheimer's disease, was evaluated by Wang et al. [71]. In their study, treatment with resveratrol (3 *μ*M, for 24 h) reduced A*β*_1–42_-induced cell death and mitochondrial damage and significantly increased mitophagy, as shown by the increased number of acidic vesicular organelles, by the enhanced expression of LC3-II, Parkin, and Beclin-1, and by the LC3 and translocase of outer mitochondrial membrane 20 (TOMM20) colocalization [71].

## 8. Kidney Disease

Cisplatin is a chemotherapy drug that is used widely to treat different cancers including testicular, germ cell, head and neck, bladder, and lung cancer; however, acute kidney injury is an important side effect of this treatment. Different mechanisms have been identified in cisplatin-induced renal injury, such as oxidative stress, apoptosis, and mitochondrial damage of renal cells. Therefore, mitochondrial homeostasis is crucial for maintaining renal function and for counteracting kidney injury [72].

In a study by Ortega-Domínguez et al., the effect of oral administration of curcumin (200 mg/kg, for 3 days) on cisplatin-induced renal damage in rats was investigated. In this study, curcumin significantly reduced tubular epithelial cell damage and necrosis. Such a nephroprotective effect was dependent on curcumin-mediated reduction of cisplatin-induced alterations in mitochondrial dynamics and function. Accordingly, curcumin prevented the increase of FIS1 and the decrease of optic atrophy 1 protein (OPA1) and NAD+-dependent deacetylase sirtuin-3 (SIRT3), crucial regulators of mitochondrial bioenergetics. In addition, curcumin decreased the expression levels of some mitophagy-associated proteins, such as PINK1 [72].

## 9. Cardiovascular Disease

Mitochondrial function is vital to those cells with a high energy expenditure like myocardiocytes. Previous studies showed that hyperglycemia may induce myocardium hypertrophy via meddling mitochondrial normal function [73].

Berberine, the main active component of the traditional Chinese medicines Coptis Root and Cortex Phellodendri, has been used to treat diabetes for thousands of years. Beyond berberine-mediated hypoglycemic effect [74–77], also antimicrobial [78], gastroprotective [79], antioxidant, anti-inflammatory [80], antifungal [81], and antihypertensive [82] effects have been reported.

The protective action of berberine on high glucose-induced cardiomyocyte hypertrophy was evaluated by Hang et al. In their study, berberine (100 nM, for 30 min) reduced H9C2 cell hypertrophy. This effect was dependent on the improvement of mitochondrial function due to the restoration of balance between fusion and fission in mitochondrial dynamics, the promotion of mitogenesis and mitophagy. The increased clearance of damaged mitochondria was mediated by the AMPK signaling pathway [73].

*Periplaneta americana* (*P. americana*), also called cockroach, is one of the largest and oldest insect groups worldwide. It has been employed as a traditional Chinese medicine for over 2,000 years, for its beneficial effects in activating blood circulation, dissipating blood stasis, promoting digestion, and inducing diuresis. Different *P. americana* extract*-*mediated pharmacological effects have been reported such as gastric protection [83], wound healing [84], antitumor activity [85], immunomodulation [86], and antifibrotic activity [87]. The effects of *P. americana* extract on lipopolysaccharide- (LPS-) induced cardiomyocyte injury was investigated by Li et al. [88]. In their study, *P. americana* extract significantly increased the viability of H9C2 cells. The expression levels of inflammatory mediators (e.g., interleukin- (IL-) 1*β*, IL-6, and tumor necrosis factor- (TNF-) *α*) were significantly reduced in *P. americana* extract-treated H9C2 cells. In addition, *P. americana* extract exerted cytoprotective effects through the regulation of mitophagy by the PINK1/Parkin pathway. In fact, the release of LC3 and the expression of PINK1, Parkin, Bnip3l, and Beclin-1 were significantly decreased in *P. americana* extract-treated H9C2 cells [88].

## 10. Respiratory Disease

Chronic obstructive pulmonary disease (COPD), including emphysema and chronic bronchitis, refers to pathological conditions characterized by airway damage leading to progressive airflow blockage and breathing-related problems. The primary cause of COPD is exposure to cigarette smoke. Previous studies showed that cigarette smoke exposure caused mitochondrial dysfunction through the decrease of mitochondrial membrane potential and the increase of mitochondrial ROS production [89].

Quercetogetin is a polymethoxyflavone found in citrus peels [90]. Different quercetogetin-mediated pharmacological effects have been reported, such as anticarcinogenic, antiviral, anti-inflammatory, antioxidant, antithrombogenic, and antiatherogenic effects [91]. The effects of quercetogetin on cigarette smoke extract-induced apoptosis of lung epithelial cells were evaluated by Son et al. [89]. In their study, treatment of Beas-2B and NHBE human bronchial epithelial cells with quercetogetin (6.25, 12.5, 25, 50, and 100 *μ*M, for 3-16 h) inhibited apoptosis by suppressing the expression of cleaved caspase-3, caspase-8, and caspase-9 and downregulating caspase activity. In addition, quercetogetin improved mitochondrial function in human bronchial epithelial cells by decreasing mitochondrial ROS production and decreased the expression of mitophagy regulatory proteins such as p-DRP1 and PINK1 [89].

## 11. Aging

The growth of the elderly population is considered a global phenomenon, and it is becoming an international challenge for healthcare systems in both developed and developing countries. Mitochondrial dysfunction contributes to aging and age-associated disease phenotypes. With aging, mitochondria undergo progressive changes in morphology, mutations in mtDNA, increase in oxidative stress, epigenetic changes in mitochondrial proteins, and defects in quality control, leading to the progressive accumulation of dysfunctional mitochondria [92].

Tomatidine is a steroidal alkaloid that has been found in the skins and leaves of tomatoes [93]. Different therapeutic effects have been described for tomatidine such as antiasthmatic [94], anti-inflammatory [95], antimicrobial [96, 97], and anticancer [98] effects. In a study by Fang et al., the effects of tomatidine (0, 25, and 50 *μ*M) on lifespan and healthspan of N2 (wild type) *Caenorhabditis elegans* (*C. elegans*) were examined. In this study, tomatidine extended lifespan and improved many *C. elegans* behaviors related to healthspan, including pharyngeal pumping and swimming movement. These beneficial tomatidine-mediated effects were due to a reduced percentage of severely damaged muscle cells. In fact, tomatidine was reported to reduce muscle cell stress by maintaining mitochondrial homeostasis and inducing mitophagy through the activation of the skinhead-1 protein (SKN-1)/nuclear factor erythroid 2- (NFE2-) related factor 2 (Nrf2) pathway [92].

## 12. Conclusion

**Figure 2:**
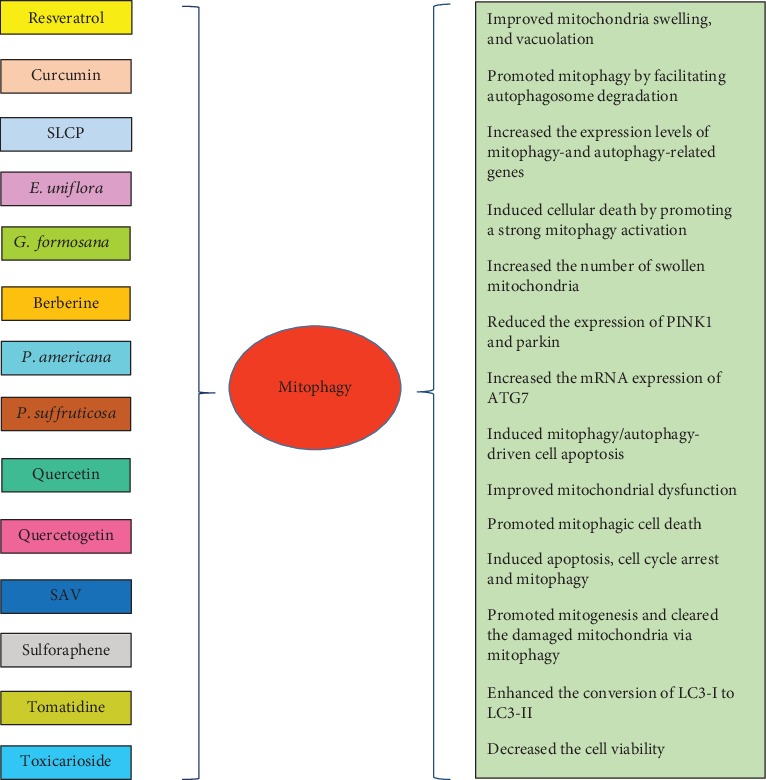
Effects of natural compounds on mitophagy.

**Figure 3:**
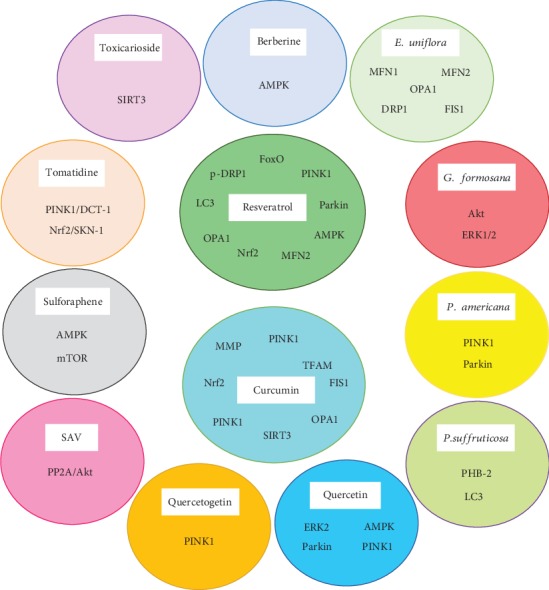
Specific molecular targets of natural compounds in the mitophagy pathway.

In this review, the effects of different natural products on various aspects of mitochondrial biology, such as mitochondrial biogenesis, membrane potential regulation, ROS production, and mitophagy, were discussed. Overall, the regulatory effects of natural products on mitophagy are exerted through multiple mechanisms (Figure 2) and through multiple molecular targets (Figure 3), which suggest the potential application of these agents as therapeutic agents in several pathological conditions associated with impaired mitophagy. Further experimental studies are required to demonstrate the exact mechanisms of action of these natural compounds and to elucidate their potential clinical applications more clearly.

## Figures and Tables

**Table 1:** Studies reporting the effects of natural compounds on mitophagy.

Natural product	Dose	Experimental model	Effect	Ref.
Resveratrol	100 mg/kg	Diquat-challenged piglets	Induced mitophagy	[8]
	0, 5, 50, and 500 mg/kg	*mdx* mice	Induced mitophagy	[33]
	0.04, 0.4, and 4 g/kg	*mdx* mice	Induced mitophagy	[34]
	1, 10, and 50 *μ*M	GRX cells	Induced mitophagy	[14]
	0.001-1000 *μ*M	MDA-MB-231, MCF-7, SiHa, HeLa, Saos-2, HUVEC cells	Induced mitophagy	[35]
	Diet containing 0.04% resveratrol	Streptozocin-induced diabetes in mice	Inhibited mitophagy	[70]
	3 *μ*M	A*β*_1–42_-treated PC12 cells	Induced mitophagy	[71]
	0, 25, and 50 *μ*mol/l	PD solution-induced peritoneal inflammatory injury in HPMCs	Induced mitophagy	[15]
Curcumin	10 *μ*M	Nasopharyngeal carcinoma CNE2 cells	Induced mitophagy	[48]
	200 mg/kg	Cisplatin-induced renal damage in rats	Inhibited mitophagy	[72]
	25 *μ*M	Human hepatoma-derived Huh-7 cells	Induced mitophagy	[49]
	25 *μ*M	U-87MG, GL261, F98, C6-glioma, and N2a cells	Induced autophagy Inhibited mitophagy	[50]
SLCP				
*E. uniflora*	5, 50, and 100 *μ*g/ml	GRX cells	Induced autophagy	[22]
*G. formosana*	0, 15, 30, 45, 60, 75, 90, 105 *μ*g/ml	HeLa, HepG2, and MCF7 cells	Induced autophagy	[54]
Berberine	100 nM	H9C2 cell line	Induced mitophagy	[73]
*P. americana*	0.25, 0.5, 1.0, 2.0, and 4.0 mg/ml	LPS-induced injury in H2C9 cells	Induced mitophagy	[88]
*P. suffruticosa*	25–2500 *μ*g/ml	PANC1, AsPC1, and BxPC3 cells	Induced mitophagy	[60]
Quercetin	100 mg/kg	Ethanol-induced hepatic damage in mice	Induced mitophagy	[27]
	100 mg/kg	HFD-induced hepatic steatosis in mice	Induced mitophagy	[28]
	100 *μ*M	HepG2 cell		
Quercetogetin	6.25, 12.5, 25, 50, and 100 *μ*M	Cigarette smoke extract-induced lung epithelial cell injury	Inhibited mitophagy	[89]
SAV	500, 1000, and 2000 mg/kg 0.5-4.5 mg/ml	BB-induced hepatotoxicity in mice LO2 and HepG2 cells	Induced mitophagy	[32]
Sulforaphene	0-20 *μ*M	U937, HUT78, Raji, JeKo-1, and U2932 cells	Induced mitophagy	[65]
Tomatidine	0, 25, and 50 *μ*M	*C. elegans*	Induced mitophagy	[92]
Toxicarioside	0-0.8 *μ*M	A549 and H460 cells	Induced mitophagy	[69]

Abbreviations: A*β*_1–42_: amyloid beta-peptide_1-42_; Atg7: autophagy-related protein 7; BB: bromobenzene; *C. elegans: Caenorhabditis elegans*; *E. uniflora*: *Eugenia uniflora*; FFA: free fatty acid; *G. formosana*: *Gynura formosana*; HFD: high-fat diet; HPMCs: human peritoneal mesothelial cells; HUVEC: human umbilical vein endothelial cell; LPS: lipopolysaccharide; *P. americana*: *Periplaneta americana*; PD: peritoneal dialysis; *P. suffruticosa*: *Paeonia suffruticosa*; SAV: Shanxi aged vinegar; SLCP: solid lipid curcumin particles.

## References

[1] Osellame L. D., Blacker T. S., Duchen M. R. (2012). Cellular and molecular mechanisms of mitochondrial function. *Best Practice & Research Clinical Endocrinology & Metabolism*.

[2] Lewis M. R., Lewis W. H. (1915). Mitochondria (and other cytoplasmic structures) in tissue cultures. *American Journal of Anatomy*.

[3] Youle R. J., Narendra D. P. (2011). Mechanisms of mitophagy. *Nature Reviews Molecular Cell Biology*.

[4] Ding W. X., Yin X. M. (2012). Mitophagy: mechanisms, pathophysiological roles, and analysis. *Biological Chemistry*.

[5] Wang K., Klionsky D. J. (2011). Mitochondria removal by autophagy. *Autophagy*.

[6] Liu Y., Zhou J., Wang L. (2016). A cyanine dye to probe mitophagy: simultaneous detection of mitochondria and autolysosomes in live cells. *Journal of the American Chemical Society*.

[7] Gibellini L., Bianchini E., de Biasi S., Nasi M., Cossarizza A., Pinti M. (2015). Natural compounds modulating mitochondrial functions. *Evidence-based Complementary and Alternative Medicine*.

[8] Cao S., Shen Z., Wang C. (2019). Resveratrol improves intestinal barrier function, alleviates mitochondrial dysfunction and induces mitophagy in diquat challenged piglets^1^. *Food & Function*.

[9] Chang X., Heene E., Qiao F., Nick P. (2011). The phytoalexin resveratrol regulates the initiation of hypersensitive cell death in Vitis cell. *PLoS One*.

[10] Han G., Xia J., Gao J., Inagaki Y., Tang W., Kokudo N. (2015). Anti-tumor effects and cellular mechanisms of resveratrol. *Drug Discoveries & Therapeutics*.

[11] Xia N., Daiber A., Förstermann U., Li H. (2017). Antioxidant effects of resveratrol in the cardiovascular system. *British Journal of Pharmacology*.

[12] Zhao X., Cui Q., Fu Q. (2017). Antiviral properties of resveratrol against pseudorabies virus are associated with the inhibition of I*κ*B kinase activation. *Scientific Reports*.

[13] Henry L. A., Witt D. M. (2002). Resveratrol: phytoestrogen effects on reproductive physiology and behavior in female rats. *Hormones and Behavior*.

[14] Meira Martins L. A., Vieira M. Q., Ilha M. (2015). The interplay between apoptosis, mitophagy and mitochondrial biogenesis induced by resveratrol can determine activated hepatic stellate cells death or survival. *Cell Biochemistry and Biophysics*.

[15] Wu J., Li X., Zhu G., Zhang Y., He M., Zhang J. (2016). The role of resveratrol-induced mitophagy/autophagy in peritoneal mesothelial cells inflammatory injury via NLRP3 inflammasome activation triggered by mitochondrial ROS. *Experimental Cell Research*.

[16] e Santos D. N., de Souza L. L., Ferreira N. J., de Oliveira A. L. (2015). Study of supercritical extraction from Brazilian cherry seeds (*Eugenia uniflora* L.) with bioactive compounds. *Food and Bioproducts Processing*.

[17] Duke J. A. (2008). *Duke’s Handbook of Medicinal Plants of Latin America*.

[18] José L. R. M., Dayane M. d. S., James O. F. (2017). Gastroprotective effect of the aqueous fraction of hydroacetonic leaf extract of Eugenia uniflora L. (Myrtaceae)(pitanga) against several gastric ulcer models in mice. *Journal of Medicinal Plants Research*.

[19] Victoria F. N., Lenardão E. J., Savegnago L. (2012). Essential oil of the leaves of *Eugenia uniflora* L.: Antioxidant and antimicrobial properties. *Food and Chemical Toxicology*.

[20] Amorim A. C., Lima C. K., Hovell A. M., Miranda A. L., Rezende C. M. (2009). Antinociceptive and hypothermic evaluation of the leaf essential oil and isolated terpenoids from Eugenia uniflora L. (Brazilian Pitanga). *Phytomedicine*.

[21] Thambi M., Tava A., Mohanakrishnan M., Subburaj M., Pradeepkumar K. M., Shafi P. M. (2013). Composition and antimicrobial activities of the essential oil from Eugenia uniflora L. leaves growing in India. *Journal of Pharmaceutical and Biomedical Research*.

[22] Denardin C. C., Martins L. A., Parisi M. M. (2017). Autophagy induced by purple pitanga (Eugenia uniflora L.) extract triggered a cooperative effect on inducing the hepatic stellate cell death. *Cell Biology and Toxicology*.

[23] Hollman P. C., Katan M. B. (1999). Dietary flavonoids: intake, health effects and bioavailability. *Food and Chemical Toxicology*.

[24] Serban M. C., Sahebkar A., Zanchetti A. (2016). Effects of quercetin on blood pressure: a systematic review and meta-analysis of randomized controlled trials. *Journal of the American Heart Association*.

[25] Hashemzaei M., Far A. D., Yari A. (2017). Anticancer and apoptosis-inducing effects of quercetin in vitro and in vivo. *Oncology Reports*.

[26] Musthafa M. E., Akbar M., Guillemin G. (2016). *The Benefits of Natural Products for Neurodegenerative Diseases*.

[27] Yu X., Xu Y., Zhang S. (2016). Quercetin attenuates chronic ethanol-induced hepatic mitochondrial damage through enhanced mitophagy. *Nutrients*.

[28] Liu P., Lin H., Xu Y. (2018). Frataxin-mediated PINK1–Parkin-dependent mitophagy in hepatic steatosis: the protective effects of quercetin. *Molecular Nutrition & Food Research*.

[29] Liang J., Xie J., Hou L. (2016). Aroma constituents in Shanxi aged vinegar before and after aging. *Journal of Agricultural and Food Chemistry*.

[30] Xie X., Zheng Y., Liu X. (2017). Antioxidant activity of Chinese Shanxi aged vinegar and its correlation with polyphenols and flavonoids during the brewing process. *Journal of Food Science*.

[31] Xia T., Yao J., Zhang J., Zheng Y., Song J., Wang M. (2017). Protective effects of Shanxi aged vinegar against hydrogen peroxide-induced oxidative damage in LO2 cells through Nrf2-mediated antioxidant responses. *RSC Advances*.

[32] Yang L., Wang X., Yang X. (2014). Possible antioxidant mechanism of melanoidins extract from Shanxi aged vinegar in mitophagy-dependent and mitophagy-independent pathways. *Journal of Agricultural and Food Chemistry*.

[33] Kuno A., Hosoda R., Sebori R. (2018). Resveratrol Ameliorates Mitophagy Disturbance and Improves Cardiac Pathophysiology of Dystrophin-deficient *mdx* Mice. *Scientific Reports*.

[34] Sebori R., Kuno A., Hosoda R., Hayashi T., Horio Y. (2018). Resveratrol decreases oxidative stress by restoring mitophagy and improves the pathophysiology of dystrophin-deficient mdx mice. *Oxidative Medicine and Cellular Longevity*.

[35] Rodríguez-Enríquez S., Pacheco-Velázquez S. C., Marín-Hernández Á. (2019). Resveratrol inhibits cancer cell proliferation by impairing oxidative phosphorylation and inducing oxidative stress. *Toxicology and Applied Pharmacology*.

[36] Mollazadeh H., Cicero A. F. G., Blesso C. N., Pirro M., Majeed M., Sahebkar A. (2019). Immune modulation by curcumin: the role of interleukin-10. *Critical Reviews in Food Science and Nutrition*.

[37] Karimian M. S., Pirro M., Majeed M., Sahebkar A. (2017). Curcumin as a natural regulator of monocyte chemoattractant protein-1. *Cytokine & Growth Factor Reviews*.

[38] Shakeri F., Boskabady M. H. (2017). Anti-inflammatory, antioxidant, and immunomodulatory effects of curcumin in ovalbumin-sensitized rat. *BioFactors*.

[39] Abdollahi E., Momtazi A. A., Johnston T. P., Sahebkar A. (2018). Therapeutic effects of curcumin in inflammatory and immune-mediated diseases: a nature-made jack-of-all-trades?. *Journal of Cellular Physiology*.

[40] Rezaee R., Momtazi A. A., Monemi A., Sahebkar A. (2017). Curcumin: a potentially powerful tool to reverse cisplatin-induced toxicity. *Pharmacological Research*.

[41] Teymouri M., Pirro M., Johnston T. P., Sahebkar A. (2017). Curcumin as a multifaceted compound against human papilloma virus infection and cervical cancers: a review of chemistry, cellular, molecular, and preclinical features. *BioFactors*.

[42] Iranshahi M., Sahebkar A., Takasaki M., Konoshima T., Tokuda H. (2009). Cancer chemopreventive activity of the prenylated coumarin, umbelliprenin, in vivo. *European Journal of Cancer Prevention*.

[43] Sahebkar A. (2010). Molecular mechanisms for curcumin benefits against ischemic injury. *Fertility and Sterility*.

[44] Panahi Y., Kianpour P., Mohtashami R., Jafari R., Simental-Mendía L. E., Sahebkar A. (2017). Efficacy and safety of phytosomal curcumin in non-alcoholic fatty liver disease: a randomized controlled trial. *Drug Research*.

[45] Emami B., Shakeri F., Ghorani V., Boskabady M. H. (2017). Relaxant effect ofCurcuma longaon rat tracheal smooth muscle and its possible mechanisms. *Pharmaceutical Biology*.

[46] Shakeri F., Roshan N. M., Kaveh M., Eftekhar N., Boskabady M. H. (2018). Curcumin affects tracheal responsiveness and lung pathology in asthmatic rats. *Pharmacological Reports*.

[47] Shakeri F., Soukhtanloo M., Boskabady M. H. (2017). The effect of hydro-ethanolic extract of *Curcuma longa* rhizome and curcumin on total and differential WBC and serum oxidant, antioxidant biomarkers in rat model of asthma. *Iranian Journal of Basic Medical Sciences*.

[48] Wang X., Leung A. W., Luo J., Xu C. (2012). TEM observation of ultrasound-induced mitophagy in nasopharyngeal carcinoma cells in the presence of curcumin. *Experimental and Therapeutic Medicine*.

[49] Moustapha A., Pérétout P. A., Rainey N. E. (2015). Curcumin induces crosstalk between autophagy and apoptosis mediated by calcium release from the endoplasmic reticulum, lysosomal destabilization and mitochondrial events. *Cell Death Discovery*.

[50] Maiti P., Scott J., Sengupta D., al-Gharaibeh A., Dunbar G. (2019). Curcumin and solid lipid curcumin particles induce autophagy, but inhibit mitophagy and the PI3K-Akt/mTOR pathway in cultured glioblastoma cells. *International Journal of Molecular Sciences*.

[51] Xu B. Q., Yang P., Zhang Y. Q. (2015). Hypoglycemic activities of lyophilized powder ofGynura divaricataby improving antioxidant potential and insulin signaling in type 2 diabetic mice. *Food & Nutrition Research*.

[52] Ma J., Guo C., Pan Y., Lin D., Qiu L., Wen L. (2017). Antioxidant and anti-inflammatory activities of ethyl acetate extract of *Gynura formosana* (Kitam) leaves. *Experimental and Therapeutic Medicine*.

[53] Hou W. C., Lin R. D., Lee T. H., Huang Y. H., Hsu F. L., Lee M. H. (2005). The phenolic constituents and free radical scavenging activities ofGynura formosana Kiamnra. *Journal of the Science of Food and Agriculture*.

[54] Ma J. F., Wei P. F., Guo C. (2018). The ethyl acetate extract of *Gynura formosana* Kitam. leaves inhibited cervical cancer cell proliferation via induction of autophagy. *BioMed Research International*.

[55] Lin H. C., Ding H. Y., Wu Y. C. (1998). Two novel compounds fromPaeoniasuffruticosa. *Journal of Natural Products*.

[56] Zhao G. H., Shen Y. S., Ma J. B., Li F., Shi X. Q. (2007). Protection of polysaccharides-2b from mudan cortex of Paeonia suffruticosa andr on diabetic cataract in rats. *Zhongguo Zhong Yao Za Zhi*.

[57] Chan B. C., Hon K. L., Leung P. C. (2008). Traditional Chinese medicine for atopic eczema: PentaHerbs formula suppresses inflammatory mediators release from mast cells. *Journal of Ethnopharmacology*.

[58] Hu P. J., Yu J., Zeng Z. R. (2004). Chemoprevention of gastric cancer by celecoxib in rats. *Gut*.

[59] Ding H. Y., Chou T. H., Lin R. J., Chan L. P., Wang G. H., Liang C. H. (2011). Antioxidant and antimelanogenic behaviors of Paeonia suffruticosa. *Plant Food for Human Nutrition*.

[60] Liu Y. H., Weng Y. P., Tsai H. Y. (2018). Aqueous extracts of Paeonia suffruticosa modulates mitochondrial proteostasis by reactive oxygen species-induced endoplasmic reticulum stress in pancreatic cancer cells. *Phytomedicine*.

[61] Qi T., Xu F., Yan X., Li S., Li H. (2016). Sulforaphane exerts anti-inflammatory effects against lipopolysaccharide-induced acute lung injury in mice through the Nrf2/ARE pathway. *International Journal of Molecular Medicine*.

[62] Pan H., He M., Liu R., Brecha N. C., Yu A. C. H., Pu M. (2014). Sulforaphane protects rodent retinas against ischemia-reperfusion injury through the activation of the Nrf2/HO-1 antioxidant pathway. *PLoS One*.

[63] Brown R. H., Reynolds C., Brooker A., Talalay P., Fahey J. W. (2015). Sulforaphane improves the bronchoprotective response in asthmatics through Nrf2-mediated gene pathways. *Respiratory Research*.

[64] Carrasco-Pozo C., Tan K. N., Borges K. (2015). Sulforaphane is anticonvulsant and improves mitochondrial function. *Journal of Neurochemistry*.

[65] Wang H., Wang F., Wu S. (2018). Traditional herbal medicine-derived sulforaphene promotes mitophagic cell death in lymphoma cells through CRM1-mediated p62/SQSTM1 accumulation and AMPK activation. *Chemico-Biological Interactions*.

[66] Dai H. F., Gan Y. J., Que D. M., Wu J., Wen Z. C., Mei W. L. (2009). A new cytotoxic 19-nor-cardenolide from the latex of Antiaris toxicaria. *Molecules*.

[67] Shi L. S., Liao Y. R., Su M. J. (2010). Cardiac glycosides from Antiaris toxicaria with potent cardiotonic activity. *Journal of Natural Products*.

[68] Choedon T., Mathan G., Arya S., Kumar V. L., Kumar V. (2006). Anticancer and cytotoxic properties of the latex of Calotropis procera in a transgenic mouse model of hepatocellular carcinoma. *World Journal of Gastroenterology*.

[69] Huang F. Y., Sun Y., Yang Y. L. (2018). Toxicarioside H induces drug-resistant mitophagy via promoting expression of sirtuin-3 in lung cancer cells. *Oncotarget*.

[70] Wang D., Sun H., Song G. (2018). Resveratrol improves muscle atrophy by modulating mitochondrial quality control in STZ-induced diabetic mice. *Molecular Nutrition & Food Research*.

[71] Wang H., Jiang T., Li W., Gao N., Zhang T. (2018). Resveratrol attenuates oxidative damage through activating mitophagy in an _in vitro_ model of Alzheimer 's disease. *Toxicology Letters*.

[72] Ortega-Domínguez B., Aparicio-Trejo O. E., García-Arroyo F. E. (2017). Curcumin prevents cisplatin-induced renal alterations in mitochondrial bioenergetics and dynamic. *Food and Chemical Toxicology*.

[73] Hang W., He B., Chen J. (2018). Berberine ameliorates high glucose-induced cardiomyocyte injury via AMPK signaling activation to stimulate mitochondrial biogenesis and restore autophagic flux. *Frontiers in Pharmacology*.

[74] Pirro M., Francisci D., Bianconi V. (2019). NUtraceutical TReatment for hYpercholesterolemia in HIV-infected patients: the NU-TRY(HIV) randomized cross-over trial. *Atherosclerosis*.

[75] Pirro M., Mannarino M. R., Bianconi V. (2016). The effects of a nutraceutical combination on plasma lipids and glucose: A systematic review and _meta_ -analysis of randomized controlled trials. *Pharmaceutical Research*.

[76] Pirro M., Vetrani C., Bianchi C., Mannarino M. R., Bernini F., Rivellese A. A. (2017). Joint position statement on "Nutraceuticals for the treatment of hypercholesterolemia" of the Italian Society of Diabetology (SID) and of the Italian Society for the Study of Arteriosclerosis (SISA). *Nutrition, Metabolism, and Cardiovascular Diseases*.

[77] Tian X., Liu F., Li Z. (2019). Enhanced anti-diabetic effect of berberine combined with timosaponin B2 in Goto-Kakizaki rats, associated with increased variety and exposure of effective substances through intestinal absorption. *Frontiers in Pharmacology*.

[78] Peng L., Kang S., Yin Z. (2015). Antibacterial activity and mechanism of berberine against Streptococcus agalactiae. *International Journal of Clinical and Experimental Pathology*.

[79] Zhang D., Ke L., Ni Z. (2017). Berberine containing quadruple therapy for initial Helicobacter pylori eradication: an open-label randomized phase IV trial. *Medicine*.

[80] Li Z., Geng Y. N., Jiang J. D., Kong W. J. (2014). Antioxidant and anti-inflammatory activities of berberine in the treatment of diabetes mellitus. *Evidence-based Complementary and Alternative Medicine*.

[81] da Silva A. R., de Andrade Neto J. B., da Silva C. R. (2016). Berberine antifungal activity in fluconazole-resistant pathogenic yeasts: action mechanism evaluated by flow cytometry and biofilm growth inhibition in Candida spp. *Antimicrobial Agents and Chemotherapy*.

[82] Zhang M., Feng L., Li J., Chen L. (2016). Therapeutic potential and mechanisms of berberine in cardiovascular disease. *Current Pharmacology Reports*.

[83] Ma X., Hu Y., Li X. (2018). Periplaneta americana ameliorates dextran sulfate sodium-induced ulcerative colitis in rats by Keap1/Nrf-2 activation, intestinal barrier function and gut microbiota regulation. *Frontiers in Pharmacology*.

[84] Song Q., Gou Q., Xie Y., Zhang Z., Fu C. (2017). *Periplaneta americana* extracts promote skin wound healing via nuclear factor kappa B canonical pathway and extracellular signal-regulated kinase signaling. *Evidence-based Complementary and Alternative Medicine*.

[85] Zhao Y., Yang A., Tu P., Hu Z. (2017). Anti-tumor effects of the American cockroach, Periplaneta americana. *Chinese Medicine*.

[86] Duarte J. P., Silva C. E., Ribeiro P. B., Cárcamo M. C. (2019). Do dietary stresses affect the immune system of Periplaneta americana (Blattaria: Blattidae)?. *Brazilian Journal of Biology*.

[87] Li D., Li W., Chen Y. (2018). Anti-fibrotic role and mechanism of Periplaneta americana extracts in CCl4-induced hepatic fibrosis in rats. *Acta Biochimica et Biophysica Sinica*.

[88] Li J., Shi W., Zhang J., Ren L. (2019). To explore the protective mechanism of PTEN-induced kinase 1 (PINK1)/Parkin mitophagy-mediated extract of Periplaneta americana on lipopolysaccharide-induced cardiomyocyte injury. *Medical Science Monitor*.

[89] Son E. S., Kim S. H., Ryter S. W. (2018). Quercetogetin protects against cigarette smoke extract-induced apoptosis in epithelial cells by inhibiting mitophagy. *Toxicology In Vitro*.

[90] Gattuso G., Barreca D., Gargiulli C., Leuzzi U., Caristi C. (2007). Flavonoid composition of citrus juices. *Molecules*.

[91] Li S., Lo C. Y., Dushenkov S., Ho C. T. (2008). Polymethoxyflavones: chemistry, biological activity, and occurrence in orange peel. *ACS Symposium Series*.

[92] Fang E. F., Waltz T. B., Kassahun H. (2017). Tomatidine enhances lifespan and healthspan in C. *elegans* through mitophagy induction via the SKN-1/Nrf2 pathway. *Scientific Reports*.

[93] Caprioli G., Cahill M., Logrippo S., James K. (2015). Elucidation of the mass fragmentation pathways of tomatidine and *β*1-hydroxytomatine using orbitrap mass spectrometry. *Natural Product Communications*.

[94] Kuo C. Y., Huang W. C., Liou C. J., Chen L. C., Shen J. J., Kuo M. L. (2017). Tomatidine attenuates airway hyperresponsiveness and inflammation by suppressing Th2 cytokines in a mouse model of asthma. *Mediators of Inflammation*.

[95] Chiu F. L., Lin J. K. (2008). Tomatidine inhibits iNOS and COX-2 through suppression of NF-kappaB and JNK pathways in LPS-stimulated mouse macrophages. *FEBS Letters*.

[96] Chagnon F., Guay I., Bonin M. A. (2014). Unraveling the structure–activity relationship of tomatidine, a steroid alkaloid with unique antibiotic properties against persistent forms of Staphylococcus aureus. *European Journal of Medicinal Chemistry*.

[97] Dyle M. C., Ebert S. M., Cook D. P. (2014). Systems-based discovery of tomatidine as a natural small molecule inhibitor of skeletal muscle atrophy. *The Journal of Biological Chemistry*.

[98] Yan K. H., Lee L. M., Yan S. H. (2013). Tomatidine inhibits invasion of human lung adenocarcinoma cell A549 by reducing matrix metalloproteinases expression. *Chemico-Biological Interactions*.

